# Association between the incidence of varicella and meteorological conditions in Jinan, Eastern China, 2012–2014

**DOI:** 10.1186/s12879-016-1507-1

**Published:** 2016-04-22

**Authors:** Yunqing Yang, Xingyi Geng, Xiaoxue Liu, Weiru Wang, Ji Zhang

**Affiliations:** Faculty of Public Health, Shandong University, Shandong Province, 250100 P. R. China; Jinan Center for Disease Control and Prevention, Shandong Province, 250021 P. R. China

**Keywords:** Varicella, Meteorological variables, Correlation analysis

## Abstract

**Background:**

Varicella remains an important public health issue in China. In this study we explored the effect of weather conditions on the incidence of varicella in the temperate city of Jinan, Eastern China during 2012–2014 to inform public health prevention and control measures.

**Methods:**

Data on reported cases of varicella were obtained from National Notifiable Disease Report System. Meteorological data for the same time period were obtained from the Jinan Meteorological Bureau. A negative binomial regression model was used to assess the relationships between meteorological variables and the incidence of varicella. Given collinearity between average temperature and atmospheric pressure, separate models were constructed: one including average temperature without atmospheric pressure, the other including atmospheric pressure but without average temperature. Both models included relative humidity, wind velocity, rainfall, sunshine, and year as independent variables.

**Results:**

Annual incidence rates of varicella were 44.47, 53.69, and 46.81 per 100,000 for 2012, 2013, and 2014, respectively. Each increase of 100 Pa (hPa) in atmospheric pressure was estimated to be associated with an increase in weekly incidence of 3.35 % (95 % CI = 2.94–3.67 %), while a 1 °C rise in temperature was associated with a decrease of 3.44 % (95 % CI = −3.73–3.15 %) in the weekly incidence of varicella. Similarly, a 1 % rise in relative humidity corresponded to a decrease of 0.50 % or 1.00 %, a 1 h rise in sunshine corresponded to an increase of 1.10 % or 0.50 %, and a 1 mm rise in rainfall corresponded to an increase of 0.20 % or 0.30 %, in the weekly incidence of varicella cases, depending on the variable considered in the model.

**Conclusion:**

Our findings show that weather factors have a significant influence on the incidence of varicella. Meteorological conditions should be considered as important predictors of varicella incidence in Jinan, Eastern China.

## Background

Varicella (commonly knows as chickenpox) is an acute infection caused by the Varicella-zoster virus (VZV) [1]. Varicella is highly contagious with an attack rate of 90 % for close contacts of cases [2]. Primary VZV infection is characterized by a generalized pruritic vesicular rash and subsequent latency in dorsal root ganglia. Shingles is a disease caused by reactivation of VZV infection and a common complication of post-herpetic neuralgia. Although varicella is generally benign and self-limiting within a week, severe complications, including death, can occur. Immunocompromised children and adults are more likely to develop severe complications [3]. Jinan is the major city of Eastern China, with over 6.95 million registered inhabitants. In Jinan, the incidence of varicella is increasing due to airborne and contact transmission: the incidence rate was 2842 per 100,000 in 2006 [4] public health authorities.

To control and prevent varicella, in 2006 the government of Jinan introduced legislation to include varicella into the list of local reportable diseases. Physicians who diagnose suspected or confirmed varicella cases must report these cases to Jinan Centers for Disease Control and Prevention (JNCDC) through the National Notifiable Disease Report System (NNDRS). Separate from the immunization strategy for measles and polio, varicella is not incorporated into the Expanded Program on Immunization (EPI) in China. Instead, it is administered as part of a voluntary vaccination program. Although routine childhood varicella vaccination has been strongly recommended by the government, the incidence of varicella in Jinan has reached a high level in recent years. In 2011, the annual incidence of varicella was 55 per 100,000, a substantially higher incidence rate than that of Japan (1.3 per 100,000) [5], Korea (35 per 100,000) [6] and Taiwan (32 per 100,000) [7].

The increasing evidence for rapid global climate change has highlighted the need to examine the relationship between weather variability and infectious diseases incidence. However, the impact of weather fluctuations on varicella is still not well understood and published evidence is inconsistent. For example, a study in Hong Kong found no significant association between temperature and varicella incidence [8], contrary to earlier studies in the West Indies [9] that suggested that temperature was inversely correlated with the incidence of varicella. Studies from Mexico [10] and Taiwan [11] have suggested that temperature is positively associated with varicella incidence. These differences are probably due to variation in weather patterns of the study areas and the different variables considered in the models. A recent study showed that atmospheric pressure and rainfall were positively associated with varicella incidence [12], but no other study has supported these findings. Furthermore, the relationship between varicella incidence and other meteorological variables, such as wind velocity and sunshine, has not yet been estimated. Therefore, there remains an urgent need to investigate these relationships to help prediction of future occurrence and to develop early warning systems for varicella.

This study aimed to describe the epidemiology of varicella in the temperate city of Jinan for 2012–2014 and to explore the association between varicella incidence and various meteorological factors.

## Methods

### Study area

Jinan covers an area of 8177 km^2^, is situated at 36°4′ north and 117°0′ east, and has over 6.95 million registered inhabitants (from 2010 census data). Jinan is warm and semi-humid with a continental monsoon climate, characterized by drought and wind in spring, warm and rainy summers, cool and dry autumns, and dry and sunny winters. The annual mean temperature was 13.8 °C and the rainfall 685 mm.

### Varicella incidence data

Since May 2006, the Jinan Ministry of Health has categorized varicella as a Class “C” infectious disease. According to notifiable infectious disease regulation, all varicella cases are reported to JNCDC through NNDRS using a standard pro forma that includes details of name, sex, age, address, and date of symptom onset. The diagnostic criteria for varicella cases are provided in a guidebook published by the Chinese Ministry of Health [13]. The case definition includes (1) fever and characteristic rash, and/or (2) a four-fold rise in antibody titre, or antigen detected in blood, or genetic material detected by polymerase chain reaction. A recent data quality survey has demonstrated that the data are of high quality in China, with reporting completeness of 99.84 % and accuracy of the information reported of 92.76 % [14]. In this study, we used the number of daily reported varicella cases in Jinan from 1 January 2012 to 31 December 2014.

### Meteorological data

Meteorological data, including daily average temperature (°C), relative humidity (%), atmospheric pressure (hPa), wind velocity (m/s), rainfall (mm) and sunshine (h) were obtained from the Jinan Meteorological Bureau (http://data.cma.gov.cn/). Weather data were measured at a fixed-site station located in the central district of Jinan using standard meteorological instruments (barometers, pressure readings, thermometers, anemometers, actinometers, psychrometers, evaporimeters, and weather vanes). Measurements of temperature, relative humidity, atmospheric pressure, and wind velocity were taken every 3 h before a daily average was calculated. For rainfall and sunshine the daily total only was calculated.

### Statistical analysis

The annual varicella incidence rate was calculated as the total number of new cases reported between 1 January and 31 December of each year, divided by the total population of the same year. A negative binomial multivariable regression model was used to estimate the associations between meteorological variables and varicella incidence. The negative binomial distribution is a Poisson distribution with an over-dispersion term; this term acts as a random effect that allows additional gamma-distributed variance to be added to the Poisson distribution. Given that data were over-dispersed, we selected a negative binomial model rather than a Poisson model. The outcome of models was the total number of varicella cases grouped according to week of onset. Meteorological variables were modeled using a weekly average or by aggregation.

As a preliminary analysis, the Pearson product–moment correlation coefficient (*r*) was used to examine the relationship between meteorological variables. If any two variables (e.g., weather factor A and weather factor B) showed a strong correlation (*r* > 0.85), two separate negative binomial regression models were constructed: one including weather factor A but not weather factor B, the other including weather factor B but not weather factor A. Both models included all other weather factors as independent variables.

To quantify the effects of meteorological variables, influences ([e^β^ − 1]*100) were estimated, corresponding to the percentage increase in varicella incidence. The final model included only those variables that reached statistical significance in the model. However, to control for yearly fluctuation, a variable for calendar year was included in the final model irrespective of statistical significance. We used the natural log of predicted rate to examine the linearity between predictor and continuous variables. In addition, residuals were checked using Pearson’s goodness of fit. All analyses were carried out using SAS (V.8.01, SAS Institute, Cary, New Jersey, USA). *P* values < 0.05 were considered to be statistically significant.

## Results

**Fig. 1:**
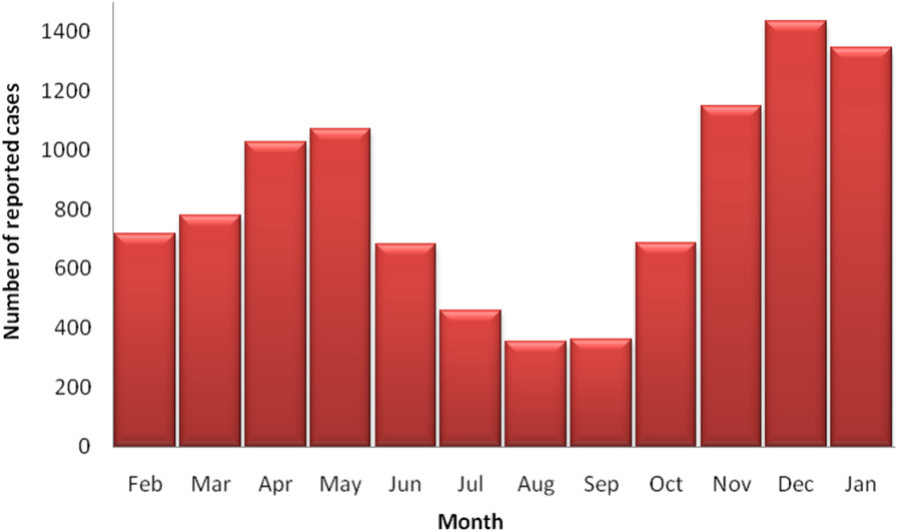
Monthly distribution of varicella reported cases in Jinan, China, 2012–2014

From 1 January 2012 to 31 December 2014, a total of 10,068 varicella cases were reported in Jinan, China, of which 56.17 % (5655) were male and 43.83 % (4413) female. The highest number of cases was in the age group of 0–5 years, which accounted for 66.16 % (6661) of the total cases. Annual incidence rates were 44.47, 53.69, and 46.81 per 100,000, in 2012, 2013, and 2014, respectively. Monthly changes in the number of cases indicated that varicella cases were detected throughout the year. A large peak in the number of cases occurred in November–January where 39.03 % of all cases were reported. A smaller peak occurred in April–May where the number of cases reported accounted for 20.84 % of total cases (Fig. 1).

**Fig. 2:**
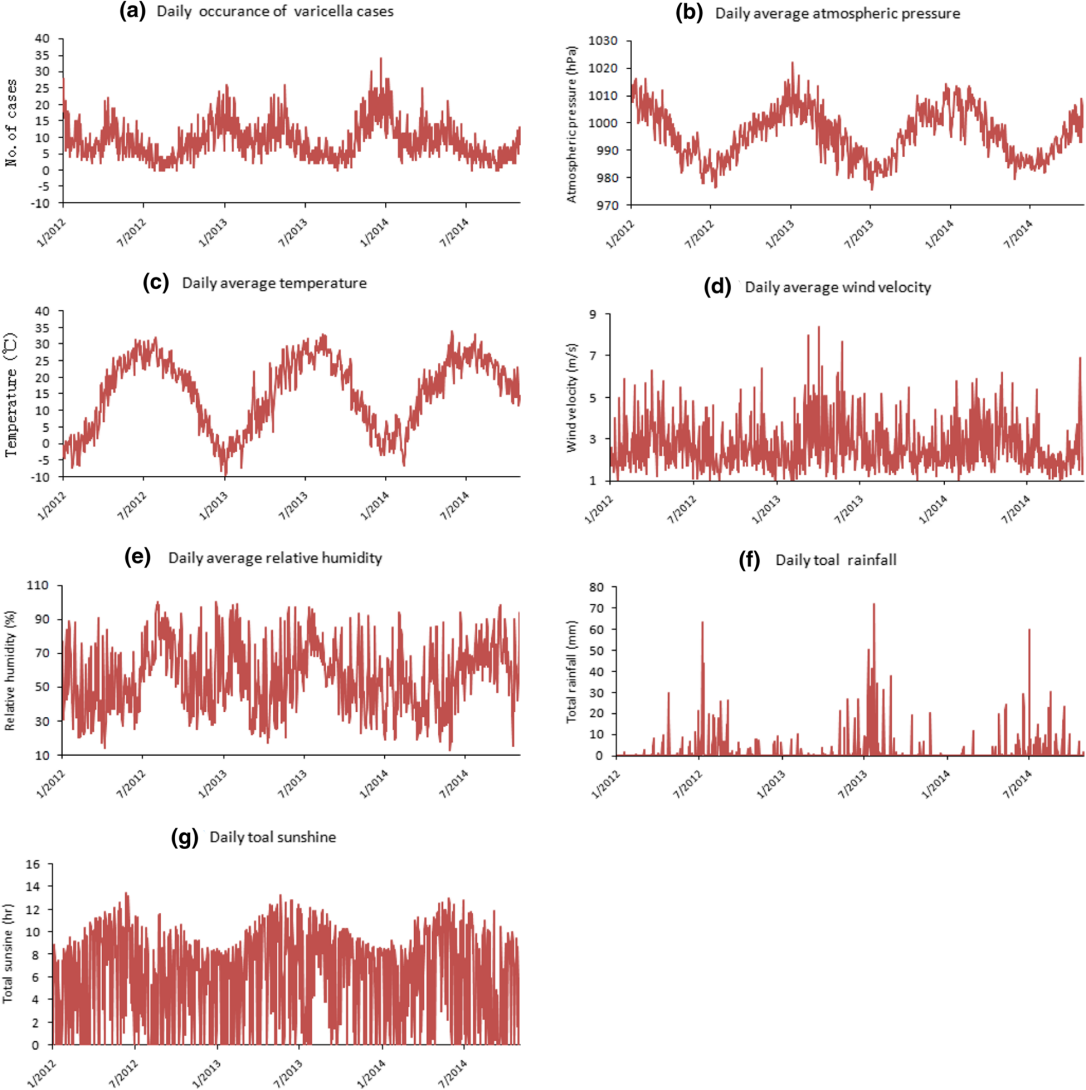
Daily distribution of **a** Varicella incidence; **b** average atmospheric pressure; **c** average temperature; **d** average wind velocity; **e** average relative humidity; **f** total rainfall; **g** total sunshine in Jinan, China, 2012–2014

As shown in Fig. 2, during the study period, the minimum and maximum temperature was −9.40 and 34.60 °C, respectively with an average temperature of 15.46 °C. Relative humidity ranged from 13.00 to 100.00 %, with an average of 56.01 %. Atmospheric pressure ranged from 857.70 hPa to 1021.80 hPa, with average of 995.79 hPa. Wind velocity ranged from 0.80 m/s to 8.40 m/s, with an average of 2.63 m/s. Daily rainfall ranged from 0 mm to 71.90 mm, with a cumulative total of 1804.25 mm for the 3-year time period. Daily sunshine ranged from 0 to 13.5 h, with a cumulative total of 6383.80 h for the 3-year time period.

Analysis revealed strong correlations (*r* = −0.87, *P* < 0.01) between average temperature and atmospheric pressure (Table 1). Therefore, to avoid potential issues with collinearity, two different models including either temperature or atmospheric pressure together with all other predictors were used to explore the relationships between temperature and atmospheric pressure and varicella incidence. In the two models, temperature (*P* < 0.01) and atmospheric pressure (*P* < 0.01) were highly significant; relative humidity, sunshine and rainfall were also statistically significant in both models (all *P* < 0.05) (Table 2).

**Table 1:** Pearson’s product–moment correlation coefficient (‘r’) matrix for meteorological variables in Jinan, China, 2012–2014

	Relative humidity	Average temperature	Wind velocity	Air pressure	Sunshine	Rainfall
Relative humidity	1.00					
Average temperature	0.13 (*P* < 0.001)	1.00				
Wind velocity	−0.35 (*P* < 0.001)	0.08 (*P* = 0.01)	1.00			
Air pressure	−0.20 (*P* < 0.001)	−0.87 (*P* < 0.001)	−0.15 (*P* < 0.001)	1.00		
Sunshine	−0.60 (*P* < 0.001)	0.21 (*P* < 0.001)	0.26 (*P* < 0.001)	−0.11(*P* < 0.001)	1.00	
Rainfall	0.37 (*P* < 0.001)	0.15 (*P* < 0.001)	−0.06 (*P* = 0.007)	−0.25 (*P* < 0.001)	−0.28 (*P* < 0.001)	1.00

**Table 2:** Negative binomial regression model of meteorological factors associated with the incidence of Varicella in Jinan, China, 2012–2014

	β	Std. error	*P*	(e^β^ − 1)^a^100 = percent increase	95 % CI for percent increase (%)	
					Lower boundary	Upper boundary
(A)						
(Intercept)	−95.1950	26.8756	0.0000	-	-	-
Year	0.0490	0.0134	0.0000	5.0220	2.3267	7.8963
Wind velocity	0.0310	0.0216	0.1490	3.1486	−1.0940	7.6807
Average temperature	−0.0350	0.0014	0.0000	−3.4395	−3.7287	−3.1493
Relative humidity	−0.0040	0.0013	0.0010	−0.3992	−0.6976	−0.1998
Sunshine	0.0100	0.0012	0.0000	1.0050	0.8032	1.3085
Rainfall	0.0020	0.0007	0.0000	0.2002	0.1001	0.4008
(B)						
(Intercept)	−59.6100	26.4741	0.0240	-	-	-
Year	0.0150	0.0131	0.2580	1.5113	−1.0940	4.1852
Wind velocity	0.0650	0.0221	0.0030	6.7159	2.2244	11.5162
Atmospheric pressure	0.0340	0.0019	0.0000	3.4585	3.0455	3.8731
Relative humidity	−0.0080	0.0013	0.0000	−0.7968	−1.0940	−0.5982
Sunshine	0.0040	0.0012	0.0000	0.4008	0.2002	0.7025
Rainfall	0.0030	0.0007	0.0000	0.3005	0.1001	0.4008
(C)						
(Intercept)	−96.5730	26.8547	0.0000	-	-	-
Year	0.0500	0.0133	0.0000	5.1271	2.4290	7.8963
Average temperature	−0.0350	0.0014	0.0000	−3.4395	−3.7287	−3.1493
Relative humidity	−0.0050	0.0012	0.0000	−0.4988	−0.6976	−0.2996
Sunshine	0.0110	0.0012	0.0000	1.1061	0.8032	1.3085
Rainfall	0.0020	0.0007	0.0000	0.2002	0.1001	0.4008
(D)						
(Intercept)	−62.3230	26.4431	0.0180	-	-	-
Year	0.0170	0.0131	0.1930	1.7145	−0.8960	4.3938
Atmospheric pressure	0.0330	0.0018	0.0000	3.3551	2.9425	3.6656
Relative humidity	−0.0100	0.0012	0.0000	−0.9950	−1.1928	−0.7968
Sunshine	0.0050	0.0011	0.0000	0.5013	0.2002	0.7025
Rainfall	0.0030	0.0007	0.0000	0.3005	0.1001	0.4008

^a^(A) and (C) were respectively preliminary and final models without atmospheric pressure. (B) and (D) were respectively preliminary and final models without average temperature

^b^CI, Confidence interval, a type of interval estimate of a population parameter

After adjusting for calendar year, each 1 hPa rise in atmospheric pressure corresponded to a 3.35 % (95 % CI = 2.94–3.67 %) rise in weekly varicella incidence, while a 1 °C rise in temperature corresponded to a decrease of 3.44 % (95 % CI = −3.73– −3.15 %), indicating a reverse effect. Likewise, depending on the variables included in the model, a 1 % rise in relative humidity corresponded to a decrease of 0.50 % or 1.00 % in the weekly incidence of varicella, a 1 h rise in sunshine corresponded to an increase of 1.10 % or 0.50 %, and a 1 mm rise in rainfall corresponded to an increase of 0.20 % or 0.30 %. All models had a good fit (Pearson’s chi-square < 0.05).

## Discussion

In recent years, varicella has accounted for significant morbidity and remains a public health issue in China [13]. During our 3-year study period, a total of 10,068 cases were reported, indicating that varicella was highly prevalent in Jinan. Although a highly effective (vaccine effectiveness 80–93 %) varicella vaccine is available and licensed in China, vaccination uptake could be improved if it was part of a universal vaccination program. However, a major obstacle is the high cost of the vaccine, which is 10 times more expensive than the rubella vaccine and 75 times more expensive than the measles vaccine in China [15]. In addition, despite the high incidence, no fatal cases have been reported, which is consistent with other regions of China [16–18], indicating that at present the epidemic status of varicella in Jinan is characterized by high morbidity but low mortality.

Similar to findings from Poland [19], Taiwan [7], and other areas of China [20], the majority of varicella cases reported in Jinan were male. This is probably due to more social activities for males whereby they are more likely to be exposed to VZV. Moreover, we found that varicella infection occurred at a very young age; the largest number of reported cases was in the age group of 0–5 years, which is contrary to the finding in subtropical country of Iraq where most cases were aged 5–14 years (65 %) [21]. Consistent with other areas of China, two peaks of varicella incidence were detected in Jinan: the major peak occurred in November–January, and a minor one in April–May. A similar situation was also observed in Korea [6], located in the same climate zone as Jinan. However, in southern Japan, which has a subtropical monsoon climate, the incidence of varicella was highest in August and lowest in winter [5]. These findings further support the role of climate factors in VZV transmission.

In recent decades, meteorological conditions have been widely studied for their potential as early warning tools to prevent climate-sensitive infectious diseases such as fecal-oral infection disease [22], malaria [23], respiratory tract infections [24], and Dengue fever [25]. Our study, demonstrates that weather factors have a significant influence on varicella incidence in Jinan. Particularly, we found that temperature was inversely correlated with varicella incidence. These findings are consistent with those of Garnett et al. who found that in the West Indies fewer varicella infections occur in tropical regions than in temperate regions [9]. In studies from India, Thailand, Sri Lanka, and other countries, varicella incidence has been reported to peak during cooler months [26–28]. A laboratory-based study has suggested that VZV seroprevalence rates in tropical climate are markedly lower in all age groups compared to temperate climates [29]. Furthermore, previous studies have shown that in vitro VZV yield and point of maximum titre were dependent on incubation temperature [30, 31]. Markus et al. demonstrated that VZV replicates more efficiently at lower temperatures [32]. In addition, our study has also indicated that high atmospheric pressure is associated with a higher incidence of varicella. This finding is in general agreement with previous findings from Hong Kong [12] that suggested atmospheric pressure might be considered an important predictor for varicella infection. However, no studies have yet been published revealing the underlying mechanism. A possible explanation might be that high atmospheric pressure is conducive to the spread of virus particles.

We found that relative humidity was inversely correlated with varicella incidence and similar findings have been observed in Hong Kong [8] and the West Indies [9]. The exact mechanism for the potential association between relative humidity and varicella incidence and transmission is unknown. It could hypothesized that in a lower relative humidity, air particles would be smaller and VZV could be suspended for longer in the air and therefore travel over a longer distance, thereby increasing transmission opportunity. Another possible explanation could be that in a dry environment, with more dry skin, patients with varicella suffer from excessive itchiness whereby more skin scratching would facilitate greater viral spread. In addition, we found that a 1 mm rise in rainfall corresponded to an increase of 0.20 % or 0.30 %, in the weekly number of varicella cases in Jinan. This finding is consistent with that of Chan et al. who showed that rainfall at lags of 2–3 days was positively associated with pediatric varicella notification in Hong Kong [12].

To the best of our knowledge, the relationship between sunshine and varicella has not been previously reported. Our study is first to investigate the effect of sunshine on varicella incidence. We found that sunshine was positively associated with varicella incidence. A possible explanation for this might be that longer periods of sunshine correspond to higher doses of solar radiation. Hervás et al. [33] found that the effects of solar radiation, directly on the host immune system and/or indirectly through vitamin D metabolism, may play a role in varicella epidemics [32]. Another possible explanation for this might be that longer periods of sunshine correspond to more time for human activities outdoors where exposure to VZV is more likely.

Some methodological limitations must be acknowledged for this study. First, surveillance data for varicella do not capture all cases in the community. This under-reporting of infections can occur anywhere in the reporting chain, from the initial decision of the patient to seek healthcare, to a failure to record the case in the disease registry due to the mildness or lack of symptoms. Second, although varicella is considered to be an easily recognizable disease [3, 34], the notification of varicella cases in our study depended solely on clinical signs, without confirming the diagnosis by microbiological or serological testing, hence resulting in potential misdiagnosis. Third, the incubation period for each case cannot be determined exactly; for this reason we chose to use weekly aggregated data of varicella cases and weekly average/aggregated meteorological data. However, the directions of these approximations are likely to be random, suggesting that our risk estimates are likely reliable. Fourth, weather information obtained from one fixed station might not be representative of the whole city. Finally, owing to this investigation being an ecological study, it is important to note that varicella transmission is multifactorial [35, 36]; besides meteorological factors, other environmental and host factors may also play a role in transmission of VZV. Our current study focused only on the meteorological factors and further studies to incorporate other environmental and host factors, including demographic factors, geographical data, population density, ethnicity, human leukocyte antigen-type predisposition, as well as behavioural data are warranted.

Climatic factors may directly influence the transmission rates of infectious diseased or be associated with changes in host susceptibility. Elucidation of the effects of weather variability on the epidemiology of infectious diseases is becoming increasingly important for disease control by public health officials and practitioners. The results of this study may aid in the prediction of epidemics and in preparation for the effects of climatic change on the epidemiology of varicella through implementation of preventive public health interventions, such as promoting good hygiene practices, temporary closure of educational institutions, active vaccination, and campaigns that include press releases and media events to encourage preventive activities. It is expected that such activities might be practically useful for preventing or limiting the spread of varicella.

## Conclusions

Varicella remains an important public health concern in Jinan. We have reported the current epidemiological situation for varicella in Jinan, characterized by high morbidity but low mortality. We found that climate parameters should be considered important predictors of varicella activity. A rise in atmospheric pressure, hours of sunshine and rainfall may increase the incidence of varicella, whereas an increase in temperature and relative humidity may reduce the incidence of varicella. Our findings provide preliminary but important information that may be useful for improving understanding of the epidemiology of varicella and help in the development of an early warning system.

### Ethics approval and consent to participate

This study was approved by the ethics committee of Jinan Center for Disease Control and Prevention (JNCDC).

### Consent for publication

Not applicable.

### Availability of data and materials

The raw data will be provided upon request by Dr. Ji Zhang (Correspondence author), Email:zhangji1967@163.com.

## Abbreviations

°C: degrees celsius
EPI: expanded program on immunization
JNCDC: Jinan Centre for Disease Control and Prevention
NNDRS: National Notifiable Disease Report System
VZV: varicella zoster virus
